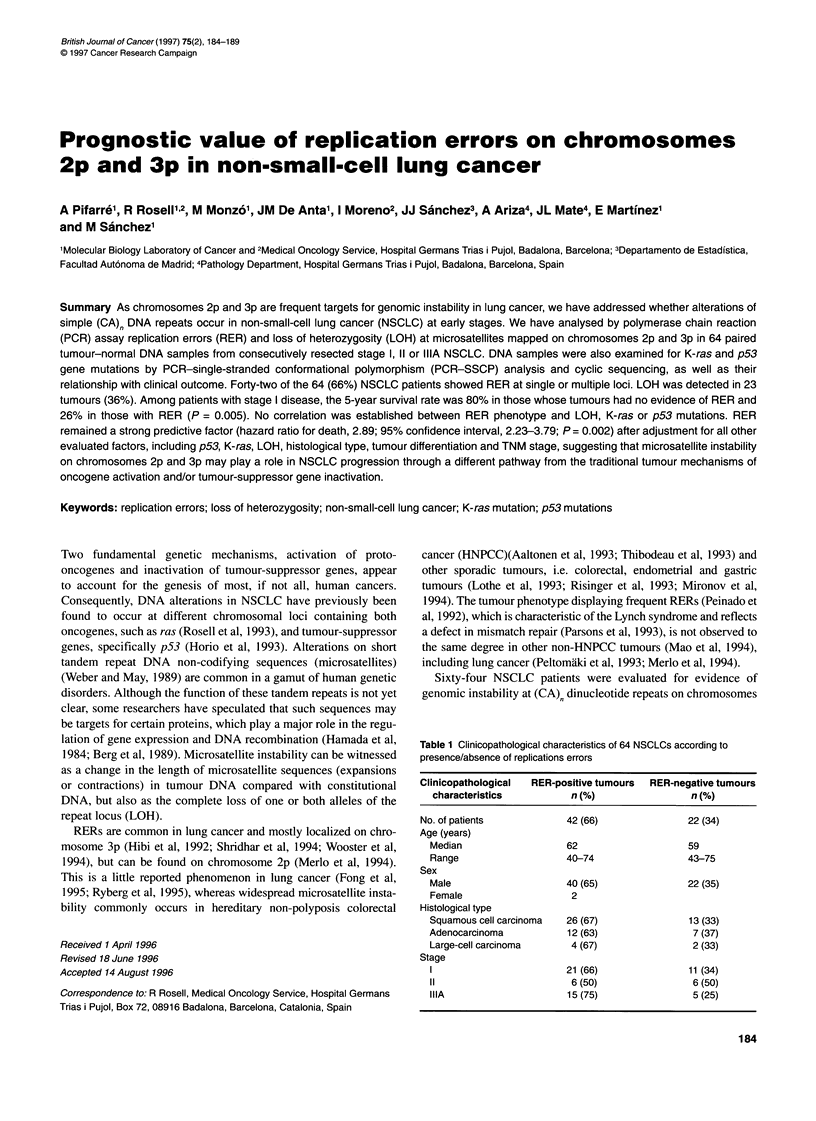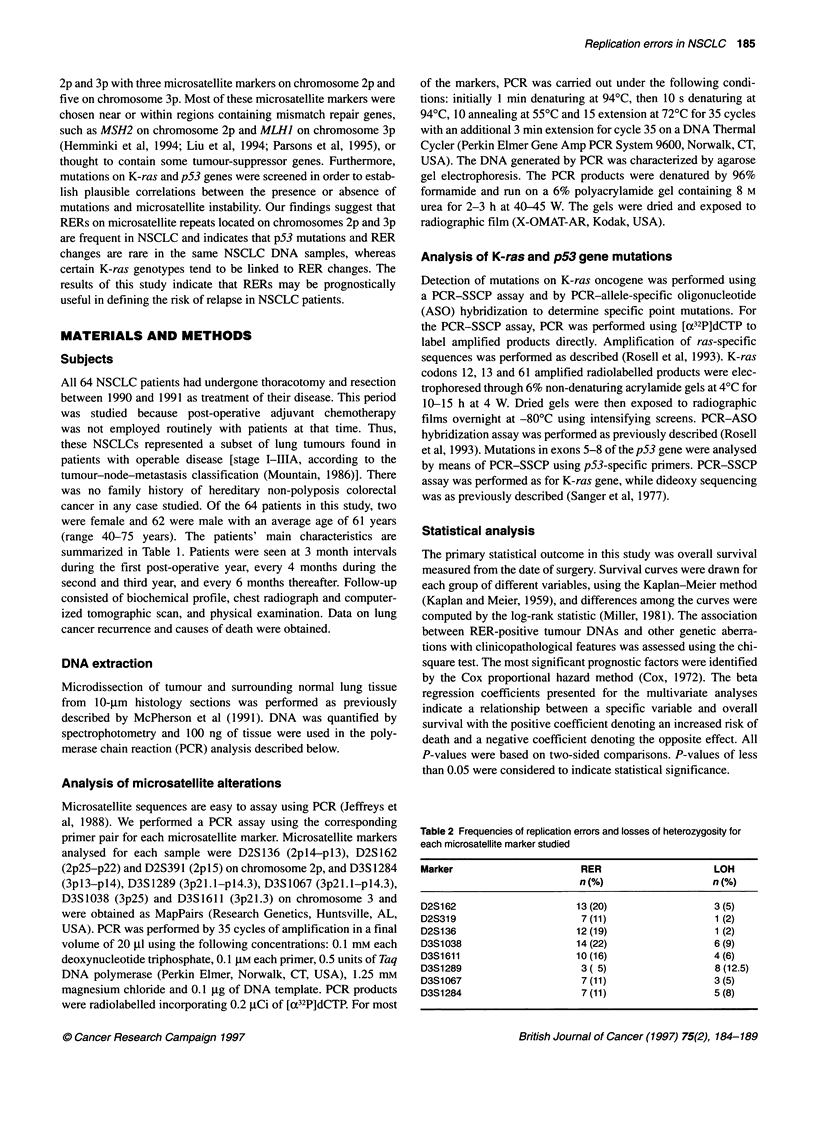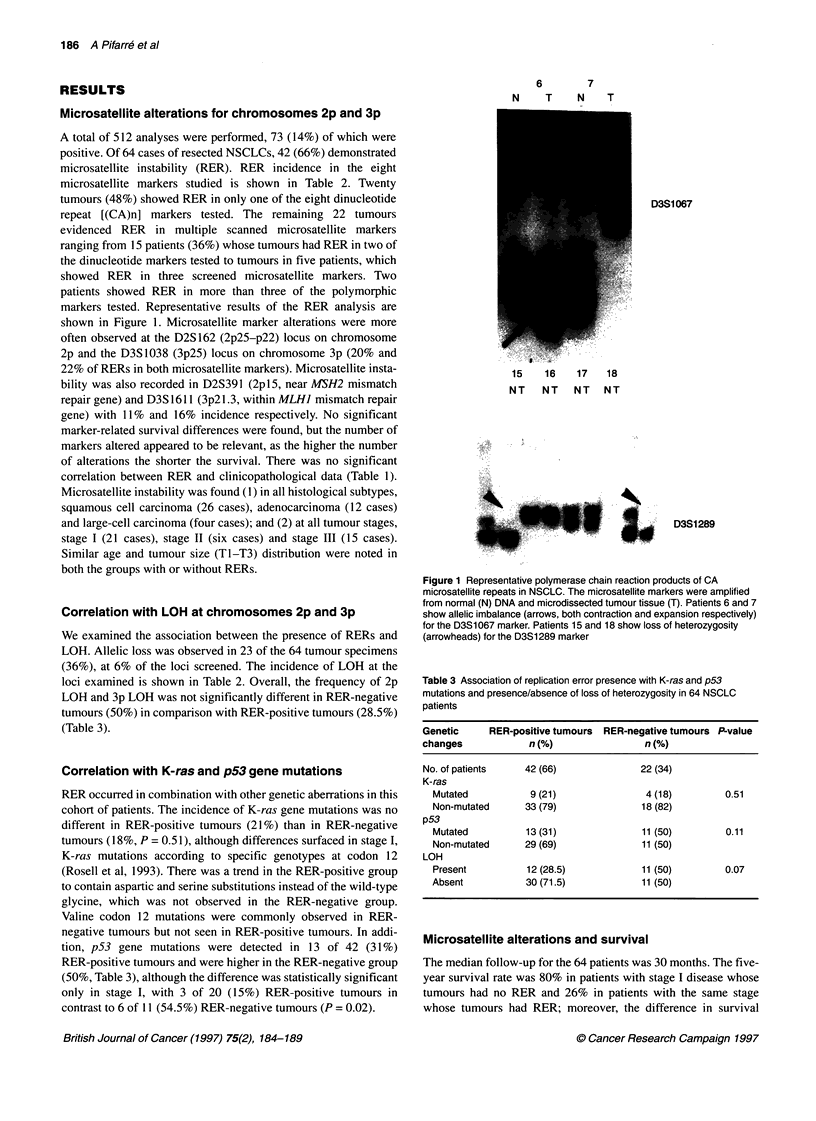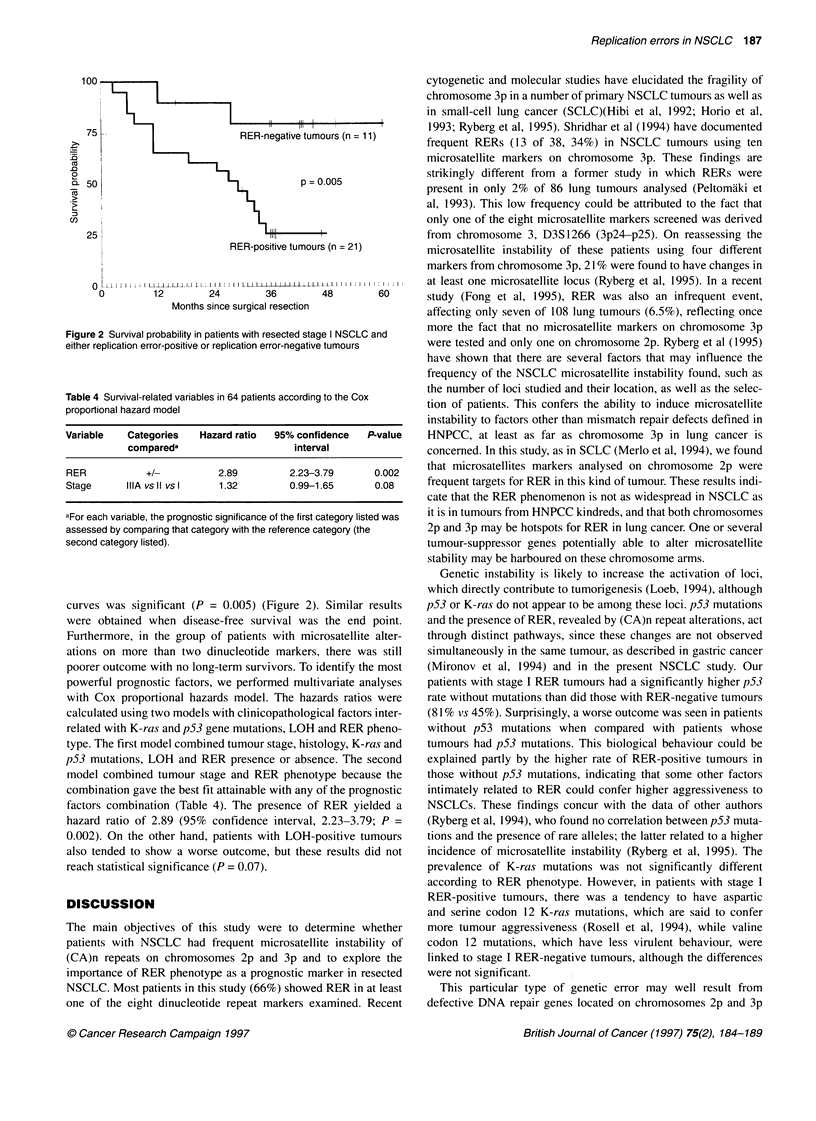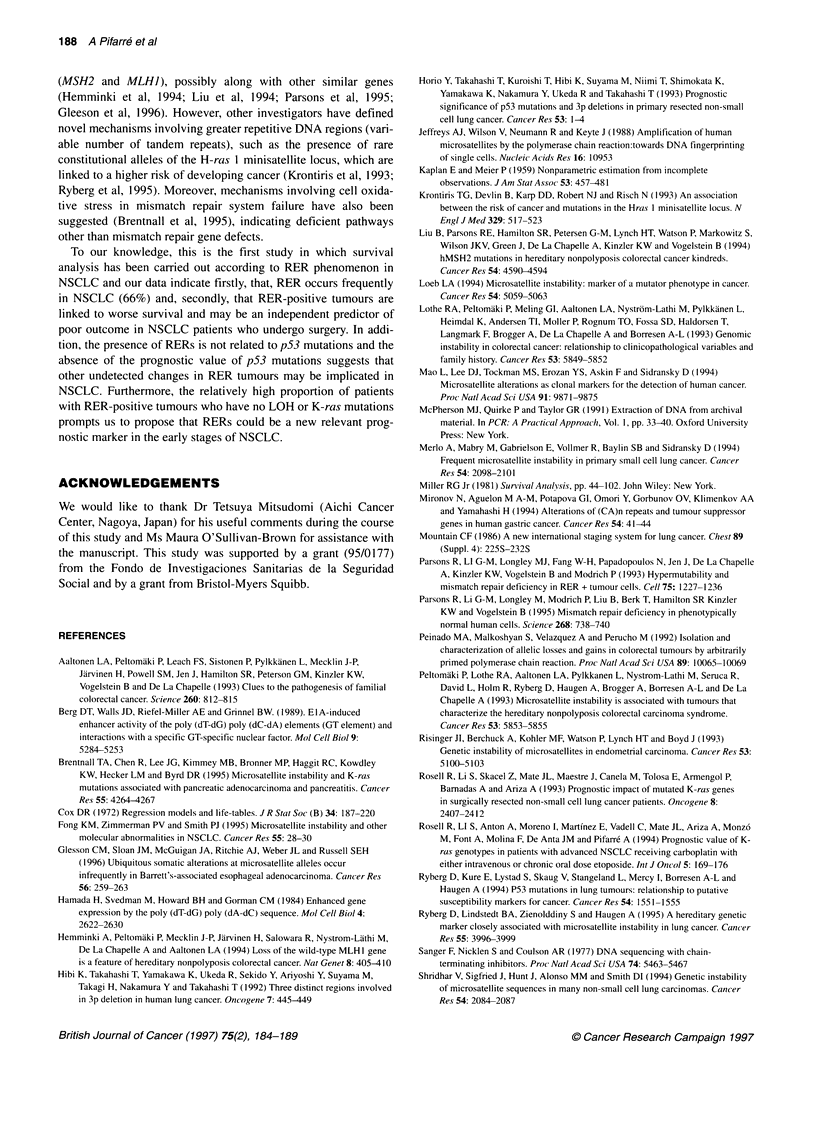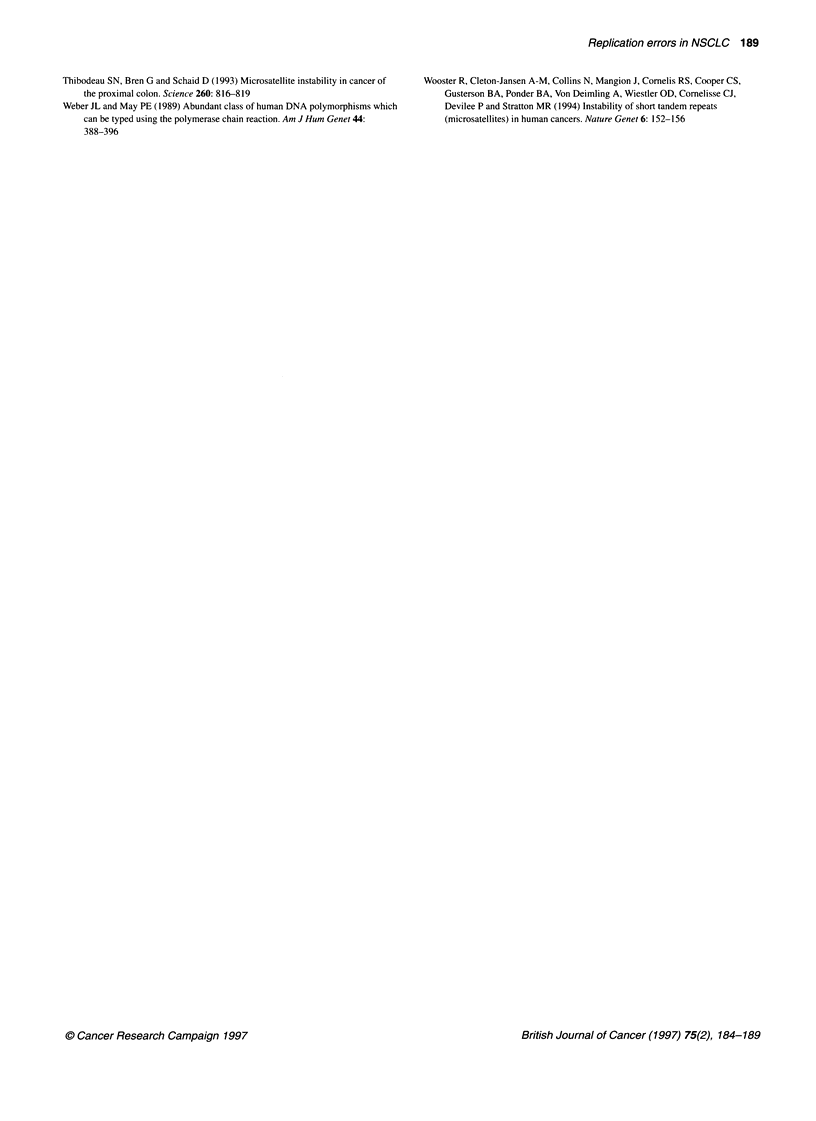# Prognostic value of replication errors on chromosomes 2p and 3p in non-small-cell lung cancer.

**DOI:** 10.1038/bjc.1997.31

**Published:** 1997

**Authors:** A. Pifarré, R. Rosell, M. Monzó, J. M. De Anta, I. Moreno, J. J. Sánchez, A. Ariza, J. L. Mate, E. Martińez, M. Sánchez

**Affiliations:** Molecular Biology Laboratory of Cancer, Hospital Germans Trias i Pujol, Badalona, Barcelona, Spain.

## Abstract

**Images:**


British Journal of Cancer (1997) 75(2), 184–189
© 1997 Cancer Research Campaign

### Prognostic value of replication errors on chromosomes 2p and 3p in non-small-cell lung cancer

A Pifarré[1], R Rosell[1,2], M Monzó[1], JM De Anta[1], I Moreno[2], JJ Sánchez[3], A Ariza[4], JL Mate[4], E Martínez[1] and M Sánchez[1]

[1]Molecular Biology Laboratory of Cancer and [2]Medical Oncology Service, Hospital Germans Trias i Pujol, Badalona, Barcelona; [3]Departamento de Estadística, Facultad Autónoma de Madrid; [4]Pathology Department, Hospital Germans Trias i Pujol, Badalona, Barcelona, Spain

**Summary** As chromosomes 2p and 3p are frequent targets for genomic instability in lung cancer, we have addressed whether alterations of simple $(CA)_n$ DNA repeats occur in non-small-cell lung cancer (NSCLC) at early stages. We have analysed by polymerase chain reaction (PCR) assay replication errors (RER) and loss of heterozygosity (LOH) at microsatellites mapped on chromosomes 2p and 3p in 64 paired tumour–normal DNA samples from consecutively resected stage I, II or IIIA NSCLC. DNA samples were also examined for K-*ras* and *p53* gene mutations by PCR–single-stranded conformational polymorphism (PCR–SSCP) analysis and cyclic sequencing, as well as their relationship with clinical outcome. Forty-two of the 64 (66%) NSCLC patients showed RER at single or multiple loci. LOH was detected in 23 tumours (36%). Among patients with stage I disease, the 5-year survival rate was 80% in those whose tumours had no evidence of RER and 26% in those with RER ($P$ = 0.005). No correlation was established between RER phenotype and LOH, K-*ras* or *p53* mutations. RER remained a strong predictive factor (hazard ratio for death, 2.89; 95% confidence interval, 2.23–3.79; $P$ = 0.002) after adjustment for all other evaluated factors, including *p53*, K-*ras*, LOH, histological type, tumour differentiation and TNM stage, suggesting that microsatellite instability on chromosomes 2p and 3p may play a role in NSCLC progression through a different pathway from the traditional tumour mechanisms of oncogene activation and/or tumour-suppressor gene inactivation.

**Keywords:** replication errors; loss of heterozygosity; non-small-cell lung cancer; K-*ras* mutation; *p53* mutations

Two fundamental genetic mechanisms, activation of proto-oncogenes and inactivation of tumour-suppressor genes, appear to account for the genesis of most, if not all, human cancers. Consequently, DNA alterations in NSCLC have previously been found to occur at different chromosomal loci containing both oncogenes, such as *ras* (Rosell et al, 1993), and tumour-suppressor genes, specifically *p53* (Horio et al, 1993). Alterations on short tandem repeat DNA non-codifying sequences (microsatellites) (Weber and May, 1989) are common in a gamut of human genetic disorders. Although the function of these tandem repeats is not yet clear, some researchers have speculated that such sequences may be targets for certain proteins, which play a major role in the regulation of gene expression and DNA recombination (Hamada et al, 1984; Berg et al, 1989). Microsatellite instability can be witnessed as a change in the length of microsatellite sequences (expansions or contractions) in tumour DNA compared with constitutional DNA, but also as the complete loss of one or both alleles of the repeat locus (LOH).

RERs are common in lung cancer and mostly localized on chromosome 3p (Hibi et al, 1992; Shridhar et al, 1994; Wooster et al, 1994), but can be found on chromosome 2p (Merlo et al, 1994). This is a little reported phenomenon in lung cancer (Fong et al, 1995; Ryberg et al, 1995), whereas widespread microsatellite instability commonly occurs in hereditary non-polyposis colorectal cancer (HNPCC)(Aaltonen et al, 1993; Thibodeau et al, 1993) and other sporadic tumours, i.e. colorectal, endometrial and gastric tumours (Lothe et al, 1993; Risinger et al, 1993; Mironov et al, 1994). The tumour phenotype displaying frequent RERs (Peinado et al, 1992), which is characteristic of the Lynch syndrome and reflects a defect in mismatch repair (Parsons et al, 1993), is not observed to the same degree in other non-HNPCC tumours (Mao et al, 1994), including lung cancer (Peltomäki et al, 1993; Merlo et al, 1994).

Sixty-four NSCLC patients were evaluated for evidence of genomic instability at $(CA)_n$ dinucleotide repeats on chromosomes

**Table 1** Clinicopathological characteristics of 64 NSCLCs according to presence/absence of replications errors

| Clinicopathological characteristics | RER-positive tumours *n* (%) | RER-negative tumours *n* (%) |
|---|---|---|
| No. of patients | 42 (66) | 22 (34) |
| Age (years) | | |
| Median | 62 | 59 |
| Range | 40–74 | 43–75 |
| Sex | | |
| Male | 40 (65) | 22 (35) |
| Female | 2 | |
| Histological type | | |
| Squamous cell carcinoma | 26 (67) | 13 (33) |
| Adenocarcinoma | 12 (63) | 7 (37) |
| Large-cell carcinoma | 4 (67) | 2 (33) |
| Stage | | |
| I | 21 (66) | 11 (34) |
| II | 6 (50) | 6 (50) |
| IIIA | 15 (75) | 5 (25) |

*Received 1 April 1996*
*Revised 18 June 1996*
*Accepted 14 August 1996*

*Correspondence to:* R Rosell, Medical Oncology Service, Hospital Germans Trias i Pujol, Box 72, 08916 Badalona, Barcelona, Catalonia, Spain

2p and 3p with three microsatellite markers on chromosome 2p and five on chromosome 3p. Most of these microsatellite markers were chosen near or within regions containing mismatch repair genes, such as *MSH2* on chromosome 2p and *MLH1* on chromosome 3p (Hemminki et al, 1994; Liu et al, 1994; Parsons et al, 1995), or thought to contain some tumour-suppressor genes. Furthermore, mutations on K-*ras* and *p53* genes were screened in order to establish plausible correlations between the presence or absence of mutations and microsatellite instability. Our findings suggest that RERs on microsatellite repeats located on chromosomes 2p and 3p are frequent in NSCLC and indicates that *p53* mutations and RER changes are rare in the same NSCLC DNA samples, whereas certain K-*ras* genotypes tend to be linked to RER changes. The results of this study indicate that RERs may be prognostically useful in defining the risk of relapse in NSCLC patients.

### MATERIALS AND METHODS

### Subjects

All 64 NSCLC patients had undergone thoracotomy and resection between 1990 and 1991 as treatment of their disease. This period was studied because post-operative adjuvant chemotherapy was not employed routinely with patients at that time. Thus, these NSCLCs represented a subset of lung tumours found in patients with operable disease [stage I–IIIA, according to the tumour–node–metastasis classification (Mountain, 1986)]. There was no family history of hereditary non-polyposis colorectal cancer in any case studied. Of the 64 patients in this study, two were female and 62 were male with an average age of 61 years (range 40–75 years). The patients' main characteristics are summarized in Table 1. Patients were seen at 3 month intervals during the first post-operative year, every 4 months during the second and third year, and every 6 months thereafter. Follow-up consisted of biochemical profile, chest radiograph and computerized tomographic scan, and physical examination. Data on lung cancer recurrence and causes of death were obtained.

### DNA extraction

Microdissection of tumour and surrounding normal lung tissue from 10-μm histology sections was performed as previously described by McPherson et al (1991). DNA was quantified by spectrophotometry and 100 ng of tissue were used in the polymerase chain reaction (PCR) analysis described below.

### Analysis of microsatellite alterations

Microsatellite sequences are easy to assay using PCR (Jeffreys et al, 1988). We performed a PCR assay using the corresponding primer pair for each microsatellite marker. Microsatellite markers analysed for each sample were D2S136 (2p14–p13), D2S162 (2p25–p22) and D2S391 (2p15) on chromosome 2p, and D3S1284 (3p13–p14), D3S1289 (3p21.1–p14.3), D3S1067 (3p21.1–p14.3), D3S1038 (3p25) and D3S1611 (3p21.3) on chromosome 3 and were obtained as MapPairs (Research Genetics, Huntsville, AL, USA). PCR was performed by 35 cycles of amplification in a final volume of 20 μl using the following concentrations: 0.1 mM each deoxynucleotide triphosphate, 0.1 μM each primer, 0.5 units of *Taq* DNA polymerase (Perkin Elmer, Norwalk, CT, USA), 1.25 mM magnesium chloride and 0.1 μg of DNA template. PCR products were radiolabelled incorporating 0.2 μCi of [$\alpha^{32}$P]dCTP. For most of the markers, PCR was carried out under the following conditions: initially 1 min denaturing at 94°C, then 10 s denaturing at 94°C, 10 annealing at 55°C and 15 extension at 72°C for 35 cycles with an additional 3 min extension for cycle 35 on a DNA Thermal Cycler (Perkin Elmer Gene Amp PCR System 9600, Norwalk, CT, USA). The DNA generated by PCR was characterized by agarose gel electrophoresis. The PCR products were denatured by 96% formamide and run on a 6% polyacrylamide gel containing 8 M urea for 2–3 h at 40–45 W. The gels were dried and exposed to radiographic film (X-OMAT-AR, Kodak, USA).

### Analysis of K-*ras* and *p53* gene mutations

Detection of mutations on K-*ras* oncogene was performed using a PCR–SSCP assay and by PCR–allele-specific oligonucleotide (ASO) hybridization to determine specific point mutations. For the PCR–SSCP assay, PCR was performed using [$\alpha^{32}$P]dCTP to label amplified products directly. Amplification of *ras*-specific sequences was performed as described (Rosell et al, 1993). K-*ras* codons 12, 13 and 61 amplified radiolabelled products were electrophoresed through 6% non-denaturing acrylamide gels at 4°C for 10–15 h at 4 W. Dried gels were then exposed to radiographic films overnight at –80°C using intensifying screens. PCR–ASO hybridization assay was performed as previously described (Rosell et al, 1993). Mutations in exons 5–8 of the *p53* gene were analysed by means of PCR–SSCP using *p53*-specific primers. PCR–SSCP assay was performed as for K-*ras* gene, while dideoxy sequencing was as previously described (Sanger et al, 1977).

### Statistical analysis

The primary statistical outcome in this study was overall survival measured from the date of surgery. Survival curves were drawn for each group of different variables, using the Kaplan–Meier method (Kaplan and Meier, 1959), and differences among the curves were computed by the log-rank statistic (Miller, 1981). The association between RER-positive tumour DNAs and other genetic aberrations with clinicopathological features was assessed using the chi-square test. The most significant prognostic factors were identified by the Cox proportional hazard method (Cox, 1972). The beta regression coefficients presented for the multivariate analyses indicate a relationship between a specific variable and overall survival with the positive coefficient denoting an increased risk of death and a negative coefficient denoting the opposite effect. All *P*-values were based on two-sided comparisons. *P*-values of less than 0.05 were considered to indicate statistical significance.

**Table 2** Frequencies of replication errors and losses of heterozygosity for each microsatellite marker studied

| Marker | RER *n* (%) | LOH *n* (%) |
|---|---|---|
| D2S162 | 13 (20) | 3 (5) |
| D2S319 | 7 (11) | 1 (2) |
| D2S136 | 12 (19) | 1 (2) |
| D3S1038 | 14 (22) | 6 (9) |
| D3S1611 | 10 (16) | 4 (6) |
| D3S1289 | 3 ( 5) | 8 (12.5) |
| D3S1067 | 7 (11) | 3 (5) |
| D3S1284 | 7 (11) | 5 (8) |

© Cancer Research Campaign 1997

### RESULTS

### Microsatellite alterations for chromosomes 2p and 3p

A total of 512 analyses were performed, 73 (14%) of which were positive. Of 64 cases of resected NSCLCs, 42 (66%) demonstrated microsatellite instability (RER). RER incidence in the eight microsatellite markers studied is shown in Table 2. Twenty tumours (48%) showed RER in only one of the eight dinucleotide repeat [(CA)n] markers tested. The remaining 22 tumours evidenced RER in multiple scanned microsatellite markers ranging from 15 patients (36%) whose tumours had RER in two of the dinucleotide markers tested to tumours in five patients, which showed RER in three screened microsatellite markers. Two patients showed RER in more than three of the polymorphic markers tested. Representative results of the RER analysis are shown in Figure 1. Microsatellite marker alterations were more often observed at the D2S162 (2p25–p22) locus on chromosome 2p and the D3S1038 (3p25) locus on chromosome 3p (20% and 22% of RERs in both microsatellite markers). Microsatellite instability was also recorded in D2S391 (2p15, near *MSH2* mismatch repair gene) and D3S1611 (3p21.3, within *MLH1* mismatch repair gene) with 11% and 16% incidence respectively. No significant marker-related survival differences were found, but the number of markers altered appeared to be relevant, as the higher the number of alterations the shorter the survival. There was no significant correlation between RER and clinicopathological data (Table 1). Microsatellite instability was found (1) in all histological subtypes, squamous cell carcinoma (26 cases), adenocarcinoma (12 cases) and large-cell carcinoma (four cases); and (2) at all tumour stages, stage I (21 cases), stage II (six cases) and stage III (15 cases). Similar age and tumour size (T1–T3) distribution were noted in both the groups with or without RERs.

### Correlation with LOH at chromosomes 2p and 3p

We examined the association between the presence of RERs and LOH. Allelic loss was observed in 23 of the 64 tumour specimens (36%), at 6% of the loci screened. The incidence of LOH at the loci examined is shown in Table 2. Overall, the frequency of 2p LOH and 3p LOH was not significantly different in RER-negative tumours (50%) in comparison with RER-positive tumours (28.5%) (Table 3).

### Correlation with K-*ras* and *p53* gene mutations

RER occurred in combination with other genetic aberrations in this cohort of patients. The incidence of K-*ras* gene mutations was no different in RER-positive tumours (21%) than in RER-negative tumours (18%, $P = 0.51$), although differences surfaced in stage I, K-*ras* mutations according to specific genotypes at codon 12 (Rosell et al, 1993). There was a trend in the RER-positive group to contain aspartic and serine substitutions instead of the wild-type glycine, which was not observed in the RER-negative group. Valine codon 12 mutations were commonly observed in RER-negative tumours but not seen in RER-positive tumours. In addition, *p53* gene mutations were detected in 13 of 42 (31%) RER-positive tumours and were higher in the RER-negative group (50%, Table 3), although the difference was statistically significant only in stage I, with 3 of 20 (15%) RER-positive tumours in contrast to 6 of 11 (54.5%) RER-negative tumours ($P = 0.02$).

**Figure 1** Representative polymerase chain reaction products of CA microsatellite repeats in NSCLC. The microsatellite markers were amplified from normal (N) DNA and microdissected tumour tissue (T). Patients 6 and 7 show allelic imbalance (arrows, both contraction and expansion respectively) for the D3S1067 marker. Patients 15 and 18 show loss of heterozygosity (arrowheads) for the D3S1289 marker

**Table 3** Association of replication error presence with K-*ras* and *p53* mutations and presence/absence of loss of heterozygosity in 64 NSCLC patients

| Genetic changes | RER-positive tumours *n* (%) | RER-negative tumours *n* (%) | *P*-value |
|---|---|---|---|
| No. of patients | 42 (66) | 22 (34) | |
| K-*ras* | | | |
| Mutated | 9 (21) | 4 (18) | 0.51 |
| Non-mutated | 33 (79) | 18 (82) | |
| *p53* | | | |
| Mutated | 13 (31) | 11 (50) | 0.11 |
| Non-mutated | 29 (69) | 11 (50) | |
| LOH | | | |
| Present | 12 (28.5) | 11 (50) | 0.07 |
| Absent | 30 (71.5) | 11 (50) | |

### Microsatellite alterations and survival

The median follow-up for the 64 patients was 30 months. The five-year survival rate was 80% in patients with stage I disease whose tumours had no RER and 26% in patients with the same stage whose tumours had RER; moreover, the difference in survival

© Cancer Research Campaign 1997

**Figure 2** Survival probability in patients with resected stage I NSCLC and either replication error-positive or replication error-negative tumours

**Table 4** Survival-related variables in 64 patients according to the Cox proportional hazard model

| Variable | Categories compared[a] | Hazard ratio | 95% confidence interval | *P*-value |
|---|---|---|---|---|
| RER | +/– | 2.89 | 2.23–3.79 | 0.002 |
| Stage | IIIA *vs* II *vs* I | 1.32 | 0.99–1.65 | 0.08 |

[a]For each variable, the prognostic significance of the first category listed was assessed by comparing that category with the reference category (the second category listed).

curves was significant ($P$ = 0.005) (Figure 2). Similar results were obtained when disease-free survival was the end point. Furthermore, in the group of patients with microsatellite alterations on more than two dinucleotide markers, there was still poorer outcome with no long-term survivors. To identify the most powerful prognostic factors, we performed multivariate analyses with Cox proportional hazards model. The hazards ratios were calculated using two models with clinicopathological factors interrelated with K-*ras* and *p53* gene mutations, LOH and RER phenotype. The first model combined tumour stage, histology, K-*ras* and *p53* mutations, LOH and RER presence or absence. The second model combined tumour stage and RER phenotype because the combination gave the best fit attainable with any of the prognostic factors combination (Table 4). The presence of RER yielded a hazard ratio of 2.89 (95% confidence interval, 2.23–3.79; $P$ = 0.002). On the other hand, patients with LOH-positive tumours also tended to show a worse outcome, but these results did not reach statistical significance ($P$ = 0.07).

### DISCUSSION

The main objectives of this study were to determine whether patients with NSCLC had frequent microsatellite instability of (CA)n repeats on chromosomes 2p and 3p and to explore the importance of RER phenotype as a prognostic marker in resected NSCLC. Most patients in this study (66%) showed RER in at least one of the eight dinucleotide repeat markers examined. Recent cytogenetic and molecular studies have elucidated the fragility of chromosome 3p in a number of primary NSCLC tumours as well as in small-cell lung cancer (SCLC)(Hibi et al, 1992; Horio et al, 1993; Ryberg et al, 1995). Shridhar et al (1994) have documented frequent RERs (13 of 38, 34%) in NSCLC tumours using ten microsatellite markers on chromosome 3p. These findings are strikingly different from a former study in which RERs were present in only 2% of 86 lung tumours analysed (Peltomäki et al, 1993). This low frequency could be attributed to the fact that only one of the eight microsatellite markers screened was derived from chromosome 3, D3S1266 (3p24–p25). On reassessing the microsatellite instability of these patients using four different markers from chromosome 3p, 21% were found to have changes in at least one microsatellite locus (Ryberg et al, 1995). In a recent study (Fong et al, 1995), RER was also an infrequent event, affecting only seven of 108 lung tumours (6.5%), reflecting once more the fact that no microsatellite markers on chromosome 3p were tested and only one on chromosome 2p. Ryberg et al (1995) have shown that there are several factors that may influence the frequency of the NSCLC microsatellite instability found, such as the number of loci studied and their location, as well as the selection of patients. This confers the ability to induce microsatellite instability to factors other than mismatch repair defects defined in HNPCC, at least as far as chromosome 3p in lung cancer is concerned. In this study, as in SCLC (Merlo et al, 1994), we found that microsatellites markers analysed on chromosome 2p were frequent targets for RER in this kind of tumour. These results indicate that the RER phenomenon is not as widespread in NSCLC as it is in tumours from HNPCC kindreds, and that both chromosomes 2p and 3p may be hotspots for RER in lung cancer. One or several tumour-suppressor genes potentially able to alter microsatellite stability may be harboured on these chromosome arms.

Genetic instability is likely to increase the activation of loci, which directly contribute to tumorigenesis (Loeb, 1994), although *p53* or K-*ras* do not appear to be among these loci. *p53* mutations and the presence of RER, revealed by (CA)n repeat alterations, act through distinct pathways, since these changes are not observed simultaneously in the same tumour, as described in gastric cancer (Mironov et al, 1994) and in the present NSCLC study. Our patients with stage I RER tumours had a significantly higher *p53* rate without mutations than did those with RER-negative tumours (81% *vs* 45%). Surprisingly, a worse outcome was seen in patients without *p*53 mutations when compared with patients whose tumours had *p53* mutations. This biological behaviour could be explained partly by the higher rate of RER-positive tumours in those without *p53* mutations, indicating that some other factors intimately related to RER could confer higher aggressiveness to NSCLCs. These findings concur with the data of other authors (Ryberg et al, 1994), who found no correlation between *p53* mutations and the presence of rare alleles; the latter related to a higher incidence of microsatellite instability (Ryberg et al, 1995). The prevalence of K-*ras* mutations was not significantly different according to RER phenotype. However, in patients with stage I RER-positive tumours, there was a tendency to have aspartic and serine codon 12 K-*ras* mutations, which are said to confer more tumour aggressiveness (Rosell et al, 1994), while valine codon 12 mutations, which have less virulent behaviour, were linked to stage I RER-negative tumours, although the differences were not significant.

This particular type of genetic error may well result from defective DNA repair genes located on chromosomes 2p and 3p

© Cancer Research Campaign 1997

(*MSH2* and *MLH1*), possibly along with other similar genes (Hemminki et al, 1994; Liu et al, 1994; Parsons et al, 1995; Gleeson et al, 1996). However, other investigators have defined novel mechanisms involving greater repetitive DNA regions (variable number of tandem repeats), such as the presence of rare constitutional alleles of the H-*ras* 1 minisatellite locus, which are linked to a higher risk of developing cancer (Krontiris et al, 1993; Ryberg et al, 1995). Moreover, mechanisms involving cell oxidative stress in mismatch repair system failure have also been suggested (Brentnall et al, 1995), indicating deficient pathways other than mismatch repair gene defects.

To our knowledge, this is the first study in which survival analysis has been carried out according to RER phenomenon in NSCLC and our data indicate firstly, that, RER occurs frequently in NSCLC (66%) and, secondly, that RER-positive tumours are linked to worse survival and may be an independent predictor of poor outcome in NSCLC patients who undergo surgery. In addition, the presence of RERs is not related to *p53* mutations and the absence of the prognostic value of *p53* mutations suggests that other undetected changes in RER tumours may be implicated in NSCLC. Furthermore, the relatively high proportion of patients with RER-positive tumours who have no LOH or K-*ras* mutations prompts us to propose that RERs could be a new relevant prognostic marker in the early stages of NSCLC.

### ACKNOWLEDGEMENTS

We would like to thank Dr Tetsuya Mitsudomi (Aichi Cancer Center, Nagoya, Japan) for his useful comments during the course of this study and Ms Maura O'Sullivan-Brown for assistance with the manuscript. This study was supported by a grant (95/0177) from the Fondo de Investigaciones Sanitarias de la Seguridad Social and by a grant from Bristol-Myers Squibb.

### REFERENCES

Aaltonen LA, Peltomäki P, Leach FS, Sistonen P, Pylkkänen L, Mecklin J-P, Järvinen H, Powell SM, Jen J, Hamilton SR, Peterson GM, Kinzler KW, Vogelstein B and De La Chapelle (1993) Clues to the pathogenesis of familial colorectal cancer. *Science* **260**: 812–815

Berg DT, Walls JD, Riefel-Miller AE and Grinnel BW. (1989). E1A-induced enhancer activity of the poly (dT-dG) poly (dC-dA) elements (GT element) and interactions with a specific GT-specific nuclear factor. *Mol Cell Biol* **9**: 5284–5253

Brentnall TA, Chen R, Lee JG, Kimmey MB, Bronner MP, Haggit RC, Kowdley KW, Hecker LM and Byrd DR (1995) Microsatellite instability and K-*ras* mutations associated with pancreatic adenocarcinoma and pancreatitis. *Cancer Res* **55**: 4264–4267

Cox DR (1972) Regression models and life-tables. *J R Stat Soc* (B) **34**: 187–220

Fong KM, Zimmerman PV and Smith PJ (1995) Microsatellite instability and other molecular abnormalities in NSCLC. *Cancer Res* **55**: 28–30

Glesson CM, Sloan JM, McGuigan JA, Ritchie AJ, Weber JL and Russell SEH (1996) Ubiquitous somatic alterations at microsatellite alleles occur infrequently in Barrett's-associated esophageal adenocarcinoma. *Cancer Res* **56**: 259–263

Hamada H, Svedman M, Howard BH and Gorman CM (1984) Enhanced gene expression by the poly (dT-dG) poly (dA-dC) sequence. *Mol Cell Biol* **4**: 2622–2630

Hemminki A, Peltomäki P, Mecklin J-P, Järvinen H, Salowara R, Nystrom-Läthi M, De La Chapelle A and Aaltonen LA (1994) Loss of the wild-type MLH1 gene is a feature of hereditary nonpolyposis colorectal cancer. *Nat Genet* **8**: 405–410

Hibi K, Takahashi T, Yamakawa K, Ukeda R, Sekido Y, Ariyoshi Y, Suyama M, Takagi H, Nakamura Y and Takahashi T (1992) Three distinct regions involved in 3p deletion in human lung cancer. *Oncogene* **7**: 445–449

Horio Y, Takahashi T, Kuroishi T, Hibi K, Suyama M, Niimi T, Shimokata K, Yamakawa K, Nakamura Y, Ukeda R and Takahashi T (1993) Prognostic significance of p53 mutations and 3p deletions in primary resected non-small cell lung cancer. *Cancer Res* **53**: 1–4

Jeffreys AJ, Wilson V, Neumann R and Keyte J (1988) Amplification of human microsatellites by the polymerase chain reaction:towards DNA fingerprinting of single cells. *Nucleic Acids Res* **16**: 10953

Kaplan E and Meier P (1959) Nonparametric estimation from incomplete observations. *J Am Stat Assoc* **53**: 457–481

Krontiris TG, Devlin B, Karp DD, Robert NJ and Risch N (1993) An association between the risk of cancer and mutations in the H*ras* 1 minisatellite locus. *N Engl J Med* **329**: 517–523

Liu B, Parsons RE, Hamilton SR, Petersen G-M, Lynch HT, Watson P, Markowitz S, Wilson JKV, Green J, De La Chapelle A, Kinzler KW and Vogelstein B (1994) hMSH2 mutations in hereditary nonpolyposis colorectal cancer kindreds. *Cancer Res* **54**: 4590–4594

Loeb LA (1994) Microsatellite instability: marker of a mutator phenotype in cancer. *Cancer Res* **54**: 5059–5063

Lothe RA, Peltomäki P, Meling GI, Aaltonen LA, Nyström-Lathi M, Pylkkänen L, Heimdal K, Andersen TI, Moller P, Rognum TO, Fossa SD, Haldorsen T, Langmark F, Brogger A, De La Chapelle A and Borresen A-L (1993) Genomic instability in colorectal cancer: relationship to clinicopathological variables and family history. *Cancer Res* **53**: 5849–5852

Mao L, Lee DJ, Tockman MS, Erozan YS, Askin F and Sidransky D (1994) Microsatellite alterations as clonal markers for the detection of human cancer. *Proc Natl Acad Sci USA* **91**: 9871–9875

McPherson MJ, Quirke P and Taylor GR (1991) Extraction of DNA from archival material. In *PCR: A Practical Approach*, Vol. 1, pp. 33–40. Oxford University Press: New York.

Merlo A, Mabry M, Gabrielson E, Vollmer R, Baylin SB and Sidransky D (1994) Frequent microsatellite instability in primary small cell lung cancer. *Cancer Res* **54**: 2098–2101

Miller RG Jr (1981) *Survival Analysis*, pp. 44–102. John Wiley: New York.

Mironov N, Aguelon M A-M, Potapova GI, Omori Y, Gorbunov OV, Klimenkov AA and Yamahashi H (1994) Alterations of (CA)n repeats and tumour suppressor genes in human gastric cancer. *Cancer Res* **54**: 41–44

Mountain CF (1986) A new international staging system for lung cancer. *Chest* **89** (Suppl. 4): 225S–232S

Parsons R, LI G-M, Longley MJ, Fang W-H, Papadopoulos N, Jen J, De La Chapelle A, Kinzler KW, Vogelstein B and Modrich P (1993) Hypermutability and mismatch repair deficiency in RER + tumour cells. *Cell* **75**: 1227–1236

Parsons R, Li G-M, Longley M, Modrich P, Liu B, Berk T, Hamilton SR Kinzler KW and Vogelstein B (1995) Mismatch repair deficiency in phenotypically normal human cells. *Science* **268**: 738–740

Peinado MA, Malkoshyan S, Velazquez A and Perucho M (1992) Isolation and characterization of allelic losses and gains in colorectal tumours by arbitrarily primed polymerase chain reaction. *Proc Natl Acad Sci USA* **89**: 10065–10069

Peltomäki P, Lothe RA, Aaltonen LA, Pylkkanen L, Nystrom-Lathi M, Seruca R, David L, Holm R, Ryberg D, Haugen A, Brogger A, Borresen A-L and De La Chapelle A (1993) Microsatellite instability is associated with tumours that characterize the hereditary nonpolyposis colorectal carcinoma syndrome. *Cancer Res* **53**: 5853–5855

Risinger JI, Berchuck A, Kohler MF, Watson P, Lynch HT and Boyd J (1993) Genetic instability of microsatellites in endometrial carcinoma. *Cancer Res* **53**: 5100–5103

Rosell R, Li S, Skacel Z, Mate JL, Maestre J, Canela M, Tolosa E, Armengol P, Barnadas A and Ariza A (1993) Prognostic impact of mutated K-*ras* genes in surgically resected non-small cell lung cancer patients. *Oncogene* **8**: 2407–2412

Rosell R, LI S, Anton A, Moreno I, Martínez E, Vadell C, Mate JL, Ariza A, Monzó M, Font A, Molina F, De Anta JM and Pifarré A (1994) Prognostic value of K-*ras* genotypes in patients with advanced NSCLC receiving carboplatin with either intravenous or chronic oral dose etoposide. *Int J Oncol* **5**: 169–176

Ryberg D, Kure E, Lystad S, Skaug V, Stangeland L, Mercy I, Borresen A-L and Haugen A (1994) P53 mutations in lung tumours: relationship to putative susceptibility markers for cancer. *Cancer Res* **54**: 1551–1555

Ryberg D, Lindstedt BA, Zienolddiny S and Haugen A (1995) A hereditary genetic marker closely associated with microsatellite instability in lung cancer. *Cancer Res* **55**: 3996–3999

Sanger F, Nicklen S and Coulson AR (1977) DNA sequencing with chain-terminating inhibitors. *Proc Natl Acad Sci USA* **74**: 5463–5467

Shridhar V, Sigfried J, Hunt J, Alonso MM and Smith DI (1994) Genetic instability of microsatellite sequences in many non-small cell lung carcinomas. *Cancer Res* **54**: 2084–2087

© Cancer Research Campaign 1997

Thibodeau SN, Bren G and Schaid D (1993) Microsatellite instability in cancer of the proximal colon. *Science* **260**: 816–819

Weber JL and May PE (1989) Abundant class of human DNA polymorphisms which can be typed using the polymerase chain reaction. *Am J Hum Genet* **44**: 388–396

Wooster R, Cleton-Jansen A-M, Collins N, Mangion J, Cornelis RS, Cooper CS, Gusterson BA, Ponder BA, Von Deimling A, Wiestler OD, Cornelisse CJ, Devilee P and Stratton MR (1994) Instability of short tandem repeats (microsatellites) in human cancers. *Nature Genet* **6**: 152–156

© Cancer Research Campaign 1997